# Assessment of Liver Fibrosis Stage and Cirrhosis Regression After Long-Term Follow-Up Following Sustained Virological Response

**DOI:** 10.3390/diagnostics16020279

**Published:** 2026-01-15

**Authors:** Lidia Canillas, Dolores Naranjo, Teresa Broquetas, Juan Sánchez, Anna Pocurull, Esther Garrido, Rosa Fernández, Xavier Forns, José A. Carrión

**Affiliations:** 1 Liver Section, Gastroenterology Department, Hospital del Mar, IMIM (Hospital del Mar Medical Research Institute), Department of Medicine and Life Sciences, Universitat Pompeu Fabra, 08003 Barcelona, Spain; lcanillas@hmar.cat (L.C.); mariateresabroquetas@bellvitgehospital.cat (T.B.); egarridop@hmar.cat (E.G.); rfernandezrodriguez@hmar.cat (R.F.); 2 Department of Gastrointestinal and Hepatobiliary Pathology, Hospital del Mar, 08003 Barcelona, Spain; dnaranjo@hmar.cat; 3 Abdominal Unit, Radiology Department, Hospital del Mar, 08003 Barcelona, Spain; jsanchezp@hmar.cat; 4 Liver Unit, Hospital Clínic, Institut D’investigacions Biomèdiques August Pi i Sunyer (IDIBAPS), Biomedical Research Net-working Center in Hepatic and Digestive Diseases (CIBEREHD), Universitat de Barcelona, 08036 Barcelona, Spain; pocurull@clinic.cat (A.P.); xforns@clinic.cat (X.F.)

**Keywords:** hepatitis C virus, fibrosis regression, non-invasive tests, VCTE-LSM, diagnostic accuracy

## Abstract

**Background/Objectives**: Previous studies have demonstrated that the cessation of liver damage after HCV cure can improve liver function, histological necroinflammation, and portal hypertension. However, scarce data about fibrosis stage or cirrhosis regression have been reported during follow-up. **Methods**: A prospective study evaluating hepatic biopsies and liver stiffness measurement by vibration-controlled transient elastography (VCTE-LSM) after the end of treatment (EOT) in patients with compensated advanced chronic liver disease (cACLD). Fibrosis was evaluated according to two semi-quantitative grading systems (METAVIR and Laennec) at 6 years after EOT (LB6) and compared with biopsies at 3 years (LB3). **Results**: Fifty-four patients with LB6 (34 with paired LB3–LB6) were included. Median (IQR) age was 53.9 (48.5–59.3), 38 (70.4%) were men, and 13 (24.1%) were HIV-coinfected. The VCTE-LSM was >15 kPa in 30 (55.6%). The LB6 (81.4 months after EOT) showed non-advanced fibrosis (F1–F2) in 12 (22.4%) patients, bridging (F3) in 26 (48.2%), and cirrhosis (F4) in 16 (29.6%): F4A in 7 (13.0%), F4B in 4 (7.4%), and F4C in 5 (9.3%). The 1-year post-EOT follow-up VCTE-LSM ≤ 8.6 kPa identifies patients without advanced fibrosis (AUROC = 0.929), with a negative predictive value of 88.9% and a positive predictive value of 95.2%. Paired biopsies showed regression in 9 (47.4%) out of 19 patients with cirrhosis: 8 (61.5%) of 13 with F4A but only 1 (16.7%) of 6 with F4B–F4C. **Conclusions**: Advanced fibrosis persists in most patients with advanced chronic liver disease after HCV eradication. Regression is possible in mild cirrhosis. However, it is a limited and slow event.

## 1. Introduction

Effective and safe treatment with direct-acting antivirals (DAAs) is well established in all stages of liver disease caused by chronic hepatitis C virus (HCV) infection. Previous studies have demonstrated that the cessation of perpetuated liver damage after HCV cure can improve liver function, histological necroinflammation, and portal hypertension [1]. As a consequence, a significant decrease in mortality risk for liver-related events (LREs) such as clinical decompensation or hepatocellular carcinoma (HCC) has been described [2,3,4,5].

The improvement in liver fibrosis after HCV eradication is not universal in patients with cirrhosis (METAVIR F4) [6] or advanced fibrosis (METAVIR F3–F4) [7,8]. A recent prospective study of our group demonstrated that advanced liver fibrosis persists even 3 years after HCV cure in 70% of patients with a previously compensated advanced chronic liver disease (cACLD) [9]. Moreover, clinically significant portal hypertension (CSPH) can persist after SVR in some cases [10,11,12]. Recent studies evaluating long-term outcomes have shown that the risks of hepatic decompensation and HCC remain over time after HCV cure in patients with cACLD [2,3,4,5]. In consonance, international guidelines recommend continuing the surveillance in patients with advanced liver fibrosis or cACLD before antiviral treatment [13,14].

The development and validation of different non-invasive tests (NITs), especially liver stiffness measurement (LSM) by vibration-controlled transient elastography (VCTE), allow the identification of patients at risk of developing LREs and the planning of their surveillance after SVR [15]. Platelet count (PLT) and VCTE-LSM are good surrogates for CSPH. The Baveno VI consensus introduced the utility of PLT and VCTE-LSM for the non-invasive assessment of CSPH [16]. In patients with active hepatitis caused by HCV, a VCTE-LSM < 15 kPa and PLT > 150 10^9^/L exclude CSPH. However, given the decrease in VCTE-LSM with HCV eradication, the criteria change to VCTE-LSM < 12 kPa and PLT > 150 10^9^/L to exclude CSPH after SVR (Baveno VII) [17]. Additionally, according to Baveno VII and the European consensus on clinical follow-up after HCV cure, patients with a post-SVR VCTE-LSM > 20 kPa may have CSPH [14,18]. However, Baveno criteria have not been evaluated to identify patients with cirrhosis after SVR.

The primary endpoint of our study was to assess the liver fibrosis stage six years after HCV eradication in patients with cACLD before antiviral treatment. The secondary objective was to evaluate variables, especially VCTE-LSM, related to the presence of non-advanced fibrosis six years after EOT. The third objective was to assess fibrosis changes and cirrhosis regression in patients with paired liver biopsies.

## 2. Materials and Methods

### 2.1. Study Design and Population

This is a prospective and observational study performed in two public hospitals in Barcelona (Hospital del Mar and Hospital Clinic).

Patients included were aged >18 years with a history of viremic HCV infection and cACLD (VCTE-LSM > 10 kPa) before antiviral treatment with DAA. Included patients achieved the end of treatment (EOT) between July 2015 and October 2018 and accepted a liver biopsy at 6 years after SVR. All patients included in our previously published study evaluating liver fibrosis stage at 3 years after SVR were asked to participate [9].

The exclusion criteria were the absence of informed consent or loss of follow-up, HBV coinfection, decompensated cirrhosis or HCC, comorbidities limiting survival, large esophageal varices, anticoagulant treatment, severe thrombopenia (<60 10^9^/L) before the liver biopsy, and a VCTE-LSM without quality criteria.

The study protocol was approved by the Ethical Committee of our institution, “Comitè Ètic d’Investigació Clínica-Parc de Salut Mar”, study reference 2017/7491/I, with date of approval 18 November 2017, following the ethical guidelines of the 1975 Declaration of Helsinki.

### 2.2. Non-Invasive Variables and Definitions

Data about liver function, assessed by blood test, abdominal ultrasonography, and VCTE-LSM, were recorded at 4 time points during the follow-up: before DAA treatment (baseline), and at one, three, and six years after EOT. Anthropometric measures, cardiovascular comorbidities (obesity, diabetes, hypertension), and alcohol intake were evaluated before antiviral treatment initiation, and after 3 and 6 years post-EOT. Harmful alcohol use was defined as consuming ≥2 or ≥3 alcoholic drinks per day for women or men, respectively [19].

The VCTE-LSM was performed using FibroScan^®^ (Echosens, Paris, France) according to the manufacturer’s recommendations and under fasting conditions. The probe (M or XL) was decided by the operator according to body mass index (BMI) and the skin–capsule distance [20]. The VCTE-LSM was considered of quality if the median value of 10 valid measurements had a ratio > 60% and an interquartile range (IQR)/VCTE-LSM < 0.3. The VCTE-LSM was recorded before DAA treatment, and at one (FU1-VCTE-LSM), three (FU3-VCTE-LSM), and six (FU6-VCTE-LSM) years after EOT. The abdominal ultrasonography evaluated the hepatic echogenicity, the nodularity of the liver surface, and the presence of splenomegaly (spleen > 12 cm) [21]. The presence and the size of esophageal varices were evaluated according to international recommendations [16].

Patients were categorized into three different groups of CSPH risk according to Baveno VI criteria [16] and Baveno VII [18] criteria [14]: (A) low risk of CSPH: VCTE-LSM < 15 kPa (before SVR) or <12 kPa (after SVR) and PLT > 150 10^9^/L; (B) intermediate risk or “grey zone”: VCTE-LSM 15–20 kPa (before SVR) or 12–20 kPa (after SVR) and PLT > 150 10^9^/L; and (C) high risk of CSPH: VCTE-LSM > 20 kPa or PLT < 150 10^9^/L (before and after SVR).

### 2.3. Histological Evaluation and Grading Systems

All patients underwent a liver biopsy 6 years (LB6) after EOT. Patients previously included [9] who accepted the LB6 underwent paired biopsies 3 (LB3) and 6 years (LB6) after HCV eradication. Liver biopsies were performed percutaneously and ultrasound-guided using a 16-G needle. The samples were formalin-fixed and paraffin-embedded, and then both hematoxylin–eosin and Masson’s Trichrome stains were evaluated [22]. Histological evaluation was performed by a double-blind reference pathologist who did not know the patient’s clinical, analytical, and VCTE-LSM data.

Only biopsy specimens with a length > 15 mm were included [22]. Necro-inflammatory activity was assessed according to the METAVIR score (A0, none; A1, mild; A2, moderate; and A3, severe), and the steatosis percentage followed the NASH CRN system (0 if <5%, 1 if 5–33%, 2 if >33–66%, and 3 if >66%) [23]. Activity grade was defined as lobule or periportal activity, whichever was higher.

Liver fibrosis was evaluated semi-quantitatively with two complementary grading systems: METAVIR and Laennec [23,24]. METAVIR graded fibrosis as 0, no definite fibrosis; 1, minimal fibrosis without septa or rare thin septum; may have portal expansion or mild sinusoidal fibrosis; 2, mild fibrosis with occasional thin septa; 3, moderate fibrosis with moderate thin septa; up to incomplete cirrhosis; and 4, cirrhosis. Non-advanced fibrosis was defined as METAVIR stages F0–F2, including no fibrosis (F0), portal or periportal fibrosis without septa (F1), and portal fibrosis with few or several septa (F2). Bridging fibrosis (METAVIR F3) was defined by the presence of numerous septa forming fibrous bridges between portal tracts and/or portal–central areas without established cirrhotic nodularity. Advanced fibrosis was defined as METAVIR stages F3–F4, corresponding to numerous septa without cirrhosis (F3) or established cirrhosis (F4) on liver biopsy.

Patients with cirrhosis were scored using Laennec as 4A, mild cirrhosis, definite or probable; 4B, moderate cirrhosis (at least two broad septa); and 4C, severe cirrhosis (at least one very broad septum or many minute nodules). The terms ‘broad septum’ and ‘very broad septum’ were defined by comparing the relative thickness of fibrous septa and the sizes of nodules.

The fibrosis changes in paired liver biopsies at 3 and 6 years after EOT were assessed as the differences in METAVIR and Laennec scores. Improvement was defined as a decrease of one or more stages in liver fibrosis, and worsening as an increase of one or more stages.

### 2.4. Sample Size and Statistical Analysis

The sample size was calculated based on Broquetas et al. [9] to determine the diagnostic accuracy of VCTE-LSM to identify cirrhosis after SVR (area under the receiver operating characteristics, AUROC = 0.747) with a null hypothesis of AUROC = 0.5, and the ratio of negative (without cirrhosis) and positive (with cirrhosis) patients after SVR (ratio = 35:41) with a type I (alpha) error level of 0.05, a type II (beta) error level of 0.10, and considering a follow-up loss of 5%. Therefore, the number of patients with liver biopsy six years after SVR was at least 54.

Categorical variables were expressed as count and percentage, and continuous variables as median and interquartile range (p25–p75) (IQR). Comparison of intra-individual data over time (before treatment, and at 3 and 6 years after EOT) was performed using the Friedman test for quantitative variables, the Cochran Q test, and the Wilcoxon test for categorical variables. Differences between patients regarding liver fibrosis stage (without advanced fibrosis F1–F2, with bridging fibrosis F3, and cirrhosis F4) were appraised with the Chi-square or U Mann–Whitney test as appropriate.

The diagnostic accuracy of VCTE-LSM to identify non-advanced fibrosis (F1–F2) 6 years after EOT was estimated using receiver operating characteristic (ROC) curves and concordance statistic (c-statistic) with the 95% confidence interval (95%CI). The reliability was considered excellent if the c-statistic was >0.9 and good if the values were between 0.7 and 0.9 [25]. We compared c-statistics using the DeLong et al. method [26]. We assessed the optimal cutoff point that maximizes the Youden index, together with specificity (Sp), sensitivity (Se), positive predictive value (PPV), and negative predictive value (NPV).

Internal validation of the ROC curve and the optimal cutoff was performed by calculating the 95% bootstrap CI of the c-statistic, and the 95% bootstrap CI of the optimal criterion was calculated using 1000 random iterations without losses, assuming the same prevalence of advanced fibrosis as observed in our cohort.

We carried out a non-parametric Wilcoxon test for categorical data and a Friedman test for continuous variables to compare biopsy characteristics in patients with paired biopsies (LB3 and LB6).

Statistical analysis was performed with STATA V18.1 (StataCorp LLC., College Station, TX, USA), IBM SPSS Statistics V26 (SPSS Inc., Chicago, IL, USA), and MedCalc V23.0.5 (MedCalc Software Ltd, Ostend, Belgium). Figures were represented with MedCalc V23.0.5 (MedCalc Software Ltd, Ostend, Belgium), GraphPad Prism V10.4.2 (GraphPad Software, Boston, MA, USA), and STATA V18.1 (StataCorp LLC., College Station, TX, USA).

## 3. Results

### 3.1. Baseline Characteristics and Follow-Up

**Figure 1:**
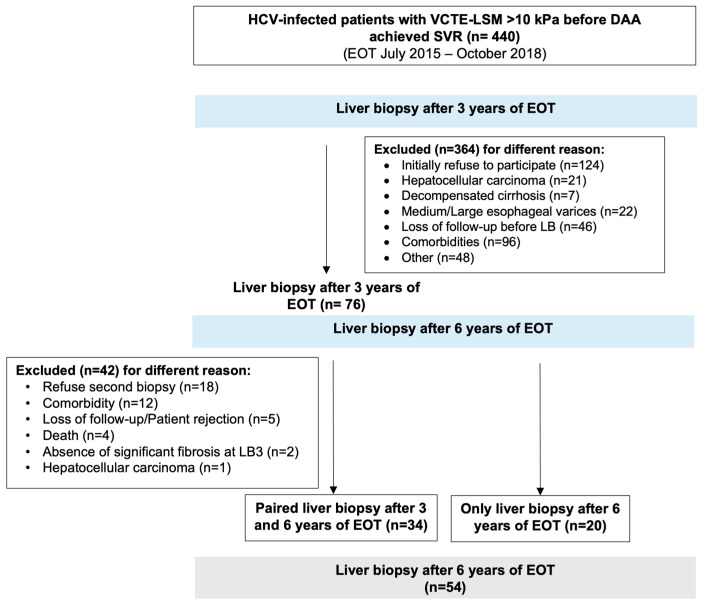
Flowchart.

We initially evaluated 440 HCV-infected patients with cACLD before antiviral treatment with DAA who achieved EOT between July 2015 and October 2018. Patients were considered to undergo a first liver biopsy after 3 years of EOT (LB3) [9] and a second one 6 years after EOT (LB6). After excluding patients for different reasons, 34 accepted paired liver biopsies (LB3 and LB6) and 20 underwent only LB6. Therefore, 54 patients with LB6 were included, and 34 (63%) of them had undergone paired liver biopsies (LB3 and LB6). The flowchart is depicted in Figure 1. No differences were found between patients with paired biopsies at 6 years after EOT.

Before the antiviral treatment, the median (IQR) age of the included patients was 53.9 (48.5–59.3) years, 38 (70.4%) were men, 14 (25.9%) were infected with HCV genotype 3, and 13 (24.1%) were coinfected with HIV. Alcohol abuse was present in 6 (11.1%) patients, and cardiovascular comorbidities in 23 (42.6%): 16 (29.6%) with obesity (BMI > 30 kg/m^2^), 12 (22.2%) with hypertension, and 10 (18.5%) with T2DM. Before treatment, VCTE-LSM was suggestive of cACLD (10–15 kPa) in 24 (44.4%) and highly suggestive of cACLD (>15 kPa) in 30 (55.6%) patients. Similarly, 28 (52.8%) patients showed a low platelet count (<150 10^9^/L). According to Baveno VI criteria (VCTE-LSM > 20 kPa and/or PLT < 150 10^9^/L) [16], 32 (59.3%) patients exhibited a high probability of CSPH, 25 (47.2%) had a spleen size > 12 cm, and 15 (out of 34 who underwent endoscopy) had esophageal varices. The baseline characteristics of included patients are shown in Table 1.

After achieving SVR, patients were followed up during a median of 81.4 (72.5–87.3) months. The proportion of patients with alcohol abuse, obesity, and T2DM was similar throughout the time (*p* = ns in all cases). However, the hypertension rate increased after SVR from 22.2% (before SVR) to 37.0% at 3 years and 42.6% at 6 years (*p* < 0.001). The liver function parameters (ALT, bilirubin, INR, albumin), the platelet count, and the VCTE-LSM improved after SVR compared to values before SVR (*p* ≤ 0.001 in all variables). Most patients normalized liver function during follow-up (Table 1).

Six years after SVR, the median (IQR) platelet count was 173 (137–225) 10^9^/L and the VCTE-LSM was 9.7 (7.5–13.6) kPa. According to Baveno VII criteria, 23 (42.6%) patients persisted with a high probability of CSPH (VCTE-LSM > 20 kPa and/or PLT< 150 10^9^/L) [18]. However, the percentage of patients with esophageal varices decreased from 27.8% before treatment to 16.7%, and the percentage of those with splenomegaly decreased from 47.2% to 14.8% at 6 years after SVR (*p* < 0.001) (Table 1).

### 3.2. Histological Evaluation 6 Years After EOT

The LB6 showed a median (IQR) length of 32 (26–35) mm and 18 (9–23) portal spaces. The inflammatory activity was null or minimal (A0–1) in 52 (96.3%) and mild (A2) in only 2 (3.7%) patients. However, 26 (48.1%) showed steatosis > 5% (Table 2). The liver fibrosis stage was evaluated semi-quantitatively using two different but complementary grading systems (METAVIR and Laennec) (Table 2). None of the patients eliminated liver fibrosis (F0). Fibrosis stage according to METAVIR was non-advanced (F1–F2) in 12 (22.2%) patients, bridging (F3) in 26 (48.2%), and cirrhosis (F4) in 16 (29.6%). Therefore, advanced fibrosis (F3–F4) persisted in 42 (77.8%) patients despite the long follow-up. In patients with cirrhosis, the Laennec classification demonstrated the persistence of F4C in 5 (9.3%), F4B in 4 (7.4%), and F4A in 7 (13.0%) patients.

### 3.3. Variables Related to Fibrosis After SVR

Differences between patients with non-advanced liver fibrosis (F1–F2) (n = 12) and those with bridging fibrosis (F3) (n = 26) and cirrhosis (F4) (n = 16) at LB6 are depicted in Table 3. No differences regarding cardiovascular comorbidities, alcohol abuse, and HIV coinfection were detected between groups (*p* = ns). Patients with advanced liver fibrosis (F3–F4) (n = 42) compared to those with non-advanced fibrosis (F1–F2) (n = 12) showed more splenomegaly (56.1% vs. 16.2%) (*p* = 0.016), PLT < 150 10^9^/L (61.9% vs. 10%) (*p* = 0.004), and high risk of CSPH according to the Baveno VI criteria (71.4% vs. 11.1%) (*p* < 0.001). In contrast, all patients with non-advanced fibrosis (F1–F2) (n = 12) showed a baseline VCTE-LSM < 20 kPa, no esophageal varices, and non-advanced cirrhosis (F4A) in LB3 (*p* < 0.01 in all cases).

**Figure 2:**
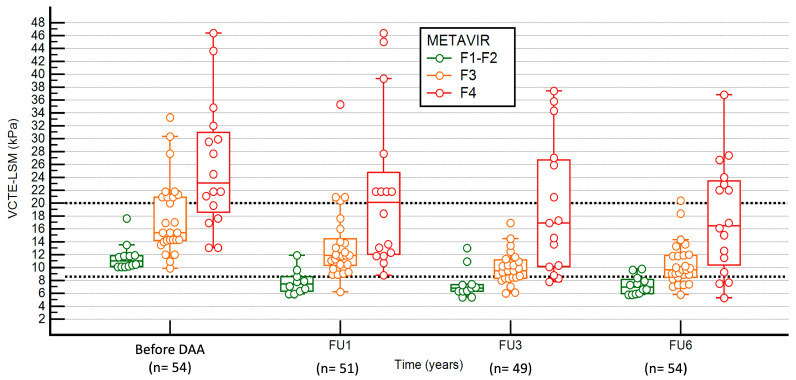
Dot plot distribution of VCTE-LSM during follow-up according to groups of liver fibrosis at 6 years post-EOT. Non-advanced fibrosis (F1–F2), bridging fibrosis (F3), and cirrhosis (F4). Dashed lines represent the VCTE-LSM cut-offs of 20 kPa and 8.6 kPa, respectively.

A total of 208 VCTE-LSM were prospectively performed and depicted in Figure 2: 54 before DAA, 51 at 1 year, 49 at 3 years, and 54 at 6 years. Patients with persistent cirrhosis (F4) (n = 16) at LB6 showed higher values of VCTE-LSM (kPa) before DAA (23.2 [18.6–31.0]), and in FU1-VCTE-LSM (20.1 [12.1–24.8]), FU3-VCTE-LSM (16.9 [10.1–27.0]), and FU6-VCTE-LSM (16.5 [10.4–23.5]) compared to those without cirrhosis (n = 38) (before DDA-VCTE-LSM of 14.3 (11.8–20.0), FU1-VCTE-LSM 11.1 (8.6–13.1), FU3-VCTE-LSM 8.6 (6.9–10.9), and FU6-VCTE-LSM 9.0 (7.3–10.2)) (*p* < 0.001 in all cases).

### 3.4. Diagnostic Accuracy of VCTE-LSM to Identify Non-Advanced Fibrosis After SVR

Due to the small number of patients, a multivariate analysis of predictive factors of non-advanced fibrosis (F1–F2) at 6 years after SVR could not be performed. Eleven (91.6%) of the 12 patients with non-advanced fibrosis (F1–F2) at LB6 showed a baseline VCTE-LSM before DAA between 10 and 15 kPa. However, 13 (31.0%) of 42 patients with advanced fibrosis (F3–F4) at LB6 also had a baseline VCTE-LSM < 15 kPa (*p* < 0.001).

**Figure 3:**
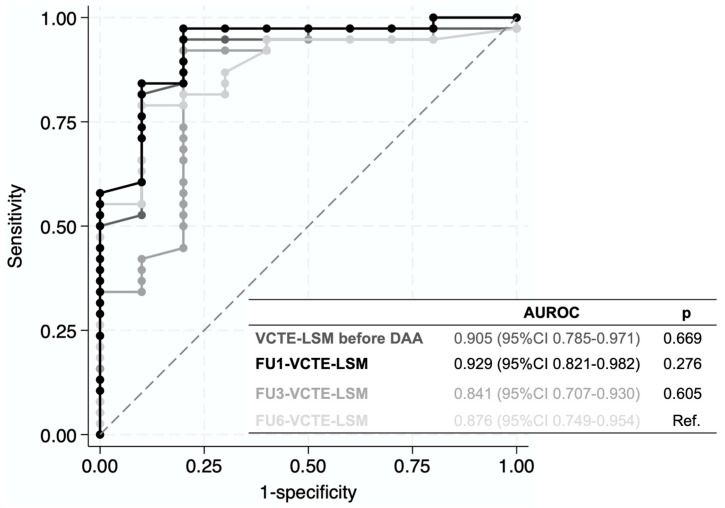
AUROC curves of VCTE-LSM to identify advanced fibrosis (F3–F4) 6 years post-EOT. The diagonal dashed line represents a non-discriminatory value, where the probability of a true positive equals that of a false positive (AUC = 0.5).

The FU1-VCTE-LSM showed the highest differences between patients with cirrhosis (F4) (20.1 [12.1–22.8] kPa), bridging fibrosis (F3) (11.9 [10.5–14.0] kPa), and without advanced fibrosis (F1–F2) (7.5 [6.3–8.6] kPa) (*p* < 0.001) at LB6 (Table 3 and Figure 2). The diagnostic accuracy of VCTE-LSM before DAA, and at 1 year, 3 years, and 6 years post-EOT is graphically shown with the ROC curves in Figure 3. The FU1-VCTE-LSM had the highest diagnostic ability to detect non-advanced fibrosis (F1–F2), with an AUROCFU1 (95%CI) of 0.929 (0.821–0.982). The AUROC (95%CI) of VCTE-LSM before DAA was 0.905 (0.785–0.971), of FU3-VCTE-LSM was 0.841 (0.707–0.930), and of FU6-VCTE-LSM was 0.876 (0.749–0.954). The internal validation of the AUROCFU1 showed a 95% bootstrap CI of 0.784–0.985.

In our cohort, the optimal cutoff of FU1-VCTE-LSM to identify advanced fibrosis (F3–F4) was 8.6 kPa, with a Se (95%CI) of 97.6% (87.1–99.9), Sp (95%CI) of 80.0% (44.4–97.5), NPV (95%CI) of 88.9% (53.0–98.3), and PPV (95%CI) of 95.2% (85.3–98.6). The internal validation of the optimal criterion showed a 95% bootstrap CI of 7.9–9.6 kPa.

### 3.5. Fibrosis Changes After Long-Term HCV Eradication

**Figure 4:**
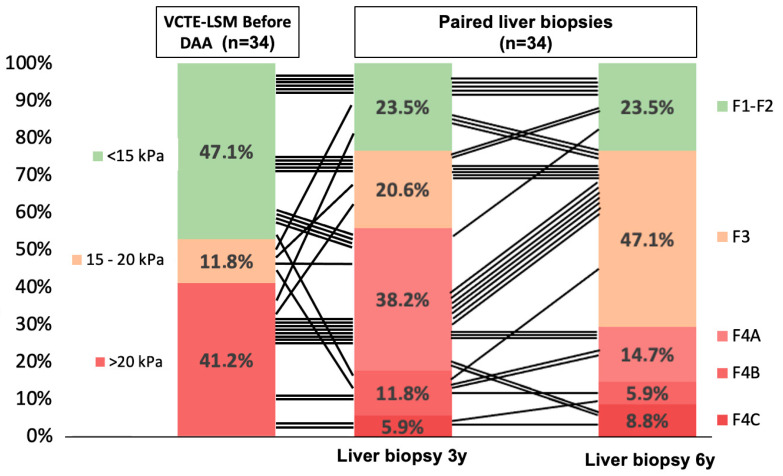
Sankey diagram shows the relation between VCTE-LSM before DAA and liver fibrosis stages (METAVIR and Laennec) at 3 and 6 years post-EOT.

Among patients with paired liver biopsies (n = 34), VCTE-LSM before DAA was <15 kPa in 16 (47.1%), from 15 to 20 kPa in 4 (11.8%), and >20 kPa in 14 (41.2%) patients (Figure 4). The fibrosis changes after EOT were evaluated in the paired liver biopsies (LB3–LB6) according to METAVIR and Laennec classifications (Table 2 and Figure 4).

All patients with a VCTE-LSM before DAA > 20 kPa showed cirrhosis (F4) at LB3. Moreover, 62.5% of those with a VCTE-LSM between 10 and 15 kPa presented advanced fibrosis (F3–F4) at LB3.

According to METAVIR, in patients with cirrhosis (F4) (n = 19) at LB3, the liver fibrosis stage regressed at LB6 in nine (47.4%) patients: eight showed F3, and only one had F2. In patients with bridging fibrosis (F3) (n = 7) at LB3, fibrosis regressed at LB6 in two (28.6%) patients to F2. In patients with non-advanced fibrosis (F1–F2) (n = 8) at LB3, the fibrosis progressed to bridging fibrosis (F3) at LB6 in three patients (37.5%) (*p* = 0.003). Therefore, 23 (88.5%) out of 26 patients with persistent advanced fibrosis (F3–F4) at LB6 have shown advanced fibrosis (F3–F4) in LB3. Consequently, the most prevalent fibrosis stage at LB6 was bridging fibrosis (F3) (n = 16, 47.1%), and only eight (23.5%) patients showed non-advanced (F1–F2) fibrosis.

The Laennec categories showed a different probability of fibrosis regression for patients with cirrhosis (n = 19) at LB3: among 13 patients with mild cirrhosis (F4A), 8 (61.5%) showed fibrosis regression compared to only 1 (16.7%) out of 6 patients with advanced cirrhosis (F4B–F4C) (*p* = 0.069).

No differences were observed between patients with fibrosis progression (from F1–F2 to F3–F4; n = 3) and those with fibrosis improvement or stability (from F3–F4 to F1–F2 or no change; n = 31) with regard to the prevalence of metabolic syndrome (hypertension, type 2 diabetes mellitus, or obesity) or alcohol consumption during follow-up. However, significant steatosis (S > 30%) at LB3 was observed in one of three patients (33.3%) with fibrosis progression, compared with none (0%) of the patients with stable or improving fibrosis (n = 31) (*p* = 0.001). Conversely, no differences were observed between patients with fibrosis improvement (n = 3) and those with stable or worsening fibrosis (n = 31) in terms of HCV genotype, HIV coinfection, metabolic syndrome prevalence, or degree of steatosis at LB3. Notably, FU3-VCTE-LSM (median [IQR]) was significantly lower in patients with fibrosis improvement (6.9 [5.4–7.3] kPa) compared with those who remained stable or experienced fibrosis worsening (10.3 [8.3–14.5] kPa; *p* = 0.011).

## 4. Discussion

Our study is the first prospective study to evaluate liver fibrosis stage and cirrhosis on paired liver biopsies in patients with cACLD after a long period following HCV eradication. In the present study, we have demonstrated that VCTE-LSM below 8.6 kPa one year after the end of antiviral treatment is an early predictor of non-advanced fibrosis. The present study has evaluated the dynamic changes in hepatic fibrosis, liver function, and VCTE-LSM, and has demonstrated that liver biopsy performed 3 years post-EOT may help identify patients with limited potential for cirrhosis regression during follow-up.

Our study has shown through liver biopsies that advanced liver fibrosis (F3–F4) persists in around 80% of patients with cACLD despite eliminating the HCV infection and a long follow-up. Only 20% of patients showed non-advanced fibrosis (F1–F2), and no patients showed resolution of liver fibrosis (F0). These results validate our previous data evaluating biopsies 3 years after EOT and emphasize the slowness of fibrosis changes in patients with advanced liver disease despite HCV eradication [9]. Around 60% of our patients exhibited a high probability of CSPH before antiviral treatment with a VCTE-LSM highly suggestive of cACLD, low platelet count, splenomegaly, and esophageal varices. Patients with persistent advanced fibrosis (F3–F4) six years after EOT showed high VCTE-LSM, splenomegaly, and low platelet count before the antiviral treatment. Therefore, the Baveno VI criteria before treatment was positive in around 70% of patients with persistent advanced liver fibrosis (F3–F4).

After SVR, the liver function (ALT, bilirubin, albumin, and INR), platelet count, and VCTE-LSM significantly improved over time. At six years following HCV eradication, more than 80% of patients achieved normal values of ALT, bilirubin, and albumin; around 60% had normal INR, platelet count, and VCTE-LSM < 10 kPa; and only 15% persisted with a high probability of CSPH defined by VCTE-LSM > 20 kPa, splenomegaly, and/or esophageal varices. Previous studies have demonstrated that HCV elimination abolishes the necroinflammation of the liver and improves liver function [9,27]. As a consequence, the values of NITs and VCTE-LSM significantly decrease after SVR.

The FU1-VCTE-LSM is a strong predictor of non-advanced fibrosis and inflammation resolution at EOT (AUROC 0.929), with diagnostic performance comparable to that of the FU6-VCTE-LSM after HCV eradication (AUROC 0.876), underscoring its clinical utility as an early risk-stratification and prognostic tool.

In contrast, the persistence of high VCTE-LSM after SVR has been associated with a higher risk of LREs. A recent multicenter study evaluating 2335 patients with cACLD, with a median time of 6 years after HCV eradication, has demonstrated that the risk of clinical decompensation in patients with FU-VCTE-LSM > 20 kPa was almost 17% at five years compared to only 0.3% in those with FU-VCTE-LSM < 10 kPa [4]. However, the capacity of FU1-VCTE-LSM to predict HCC risk has been insufficiently explored [5]. In an observational study with 1046 patients with HCV cACLD, the authors found that 45 (3.9%) of them developed HCC after a median follow-up of 45 months. The median FU1-VCTE-LSM was higher in patients who developed HCC (19.5 kPa) compared to those who did not (12.1 kPa) (*p* < 0.001). The FU1-VCTE-LSM and the delta between baseline and FU1-VCTE-LSM were predictors of HCC, but the authors did not explore the accuracy of the FU1-VCTE-LSM cutoffs [28].

More than 60% of our cohort underwent paired liver biopsies at 3 and 6 years after EOT, allowing us to evaluate changes in fibrosis after HCV eradication. Paired biopsies demonstrated that almost 50% of patients with cirrhosis (F4) at 3 years after EOT can regress, but most of them persist with bridging fibrosis (F3). Importantly, almost 90% of patients with persistent advanced fibrosis (F3–F4) at 6 years after HCV eradication had shown advanced fibrosis 3 years after EOT.

Moreover, three out of eight patients (37.5%) with non-advanced fibrosis at 3 years after EOT showed fibrosis worsening at 6 years. Significant steatosis was the only variable associated with this progression. A previously published study evaluating fibrosis changes in 15 paired liver biopsies (before and after SVR) with advanced fibrosis [7] showed that only four patients regressed to non-advanced fibrosis after a median follow-up of 2 years. A more recent study, using a digital imaging system, identified the reversibility of liver fibrosis in around 33% of paired biopsies after 12 months of follow-up [8]. However, information regarding comorbidities, alcohol abuse, antiviral treatment, and baseline liver fibrosis stage was lacking. It is important to note that an increase in VCTE-LSM after SVR does not invariably reflect fibrosis progression. Although LSM primarily reflects hepatic fibrosis, it can also be influenced by steatosis and metabolic inflammation. Emerging evidence suggests that metabolic dysfunction-associated steatotic liver disease may increase VCTE-LSM independently of fibrosis [29]. Therefore, histological assessment remains the gold standard when non-invasive tests yield discordant results [14].

Although the reversal of fibrosis appears to be an encouraging approach to the treatment of chronic liver diseases, further studies are necessary to better understand the mechanisms [30]. Advanced stages of cirrhosis can progress independently of the initial etiological agent, due to a vicious circle in which vascular injury promotes vascular obstruction and hepatocellular damage [31]. Moreover, the persistence of scar tissue can be associated, on one hand, with the maintenance of activated hepatic stellate cells, and on the other hand, inactivated hepatic stellate cells can remain more sensitive to new fibrogenic stimuli [32].

It is important to note that the Laennec categorization of cirrhosis identified patients in our cohort with a lower probability of fibrosis regression. Around 60% of patients with mild cirrhosis (Laennec F4A) at 3 years after EOT showed fibrosis regression compared to only one out of six with advanced cirrhosis (Laennec F4B–F4C). The Laennec staging system is a modification of the METAVIR system that divides cirrhosis into three groups based on the thickness of the fibrous septa and the size of the nodules. The Laennec classification was proposed to stratify patients with HCV cirrhosis and predict the development of hepatic decompensation [33]. In patients with HCV infection, higher grades of the Laennec system have been associated with higher portal hypertension, higher risk for hepatocellular carcinoma recurrence, more LREs, and worse prognosis [24,33,34]. On the other hand, the International Liver Pathology Study Group described the features of fibrosis regression, such as interrupted and floating fibrous septa, identified only in patients with mild cirrhosis of Laennec stage F4A [35]. However, the applicability of the Laennec staging system after SVR has not been well established before our study [6]. Our results suggest that performing a liver biopsy 3 years after SVR could identify patients without a long-term regression capacity if showing an advanced cirrhosis or Laennec F4B–F4C fibrosis stage.

Our study has some limitations. The lack of biopsies before antiviral therapy does not allow us to know the exact rate of cirrhosis before antiviral treatment. However, the use of validated Baveno VI criteria is an excellent approximation. Another drawback is the limited sample size, due to the low rate of biopsy acceptance by patients after HCV elimination. Nevertheless, 60% underwent paired liver biopsies at 3 and 6 years of follow-up, allowing us to validate the histological results and evaluate the fibrosis changes.

We believe that our study has notable strengths. This is probably the largest prospective study evaluating liver fibrosis stage and cirrhosis regression in paired liver biopsies after a long period following HCV eradication in patients with advanced chronic liver disease. Moreover, patients were prospectively monitored with VCTE-LSM six years after EOT, and values one year after EOT showed excellent accuracy in identifying patients without advanced fibrosis. In addition, the bootstrapping statistical method allowed us to internally validate the optimal cutoff of VCTE-LSM for predicting, one year after EOT, the presence of non-advanced fibrosis following HCV eradication.

## 5. Conclusions

In conclusion, our study demonstrates that advanced liver fibrosis persists in most patients with cACLD despite HCV eradication. However, a VCTE-LSM value below 8.6 kPa one year after the end of antiviral treatment appears to be associated with non-advanced fibrosis and shows diagnostic performance comparable to that of VCTE-LSM assessed six years after HCV eradication. While fibrosis regression may occur in patients with mild cirrhosis, it seems to be a slow and limited process and appears minimal in patients with Laennec stage F4B or F4C fibrosis.

## Figures and Tables

**Table 1:** Baseline characteristics and follow-up. Impact of HCV eradication on comorbidities, liver function, portal hypertension, VCTE-LSM, CAP, and Baveno VI and VII criteria.

	Before DAA (n = 54)	3 Years After EOT (n = 54)	6 Years After EOT (n = 54)	*p* ^1^	*p* ^2^
BMI (kg/m^2^)	26.8 (24.2–30.4)	27.2 (24.7–30.0)	26.2 (24.5–29.6)	0.286	0.189
Obesity, n (%)	16 (29.6)	12 (24.5)	13 (24.1)	0.157	1.000
T2DM, n (%)	10 (18.5)	12 (22.2)	12 (22.2)	0.157	1.000
Hypertension, n (%)	12 (22.2)	20 (37.0)	23 (42.6)	0.011	0.083
At least 1 comorbidity, n (%)	23 (42.6)	27 (54.0)	29 (53.7)	0.014	0.564
Alcohol abuse, n (%)	6 (11.1)	2 (3.7)	4 (7.4)	0.157	0.414
ALT (U/L)	59 (45–112)	23 (19–29)	24 (19–28)	<0.001	0.238
ALT > 40 U/L, n (%)	48 (90.6)	6 (11.5)	7 (13.0)	<0.001	0.782
Bilirubin (mg/dL)	0.8 (0.6–1.2)	0.5 (0.4–0.9)	0.6 (0.4–0.8)	0.007	0.668
Bilirubin > 1 mg/dL, n (%)	0 (0)	10 (19.6)	8 (14.8)	0.317	0.317
Albumin (g/dL)	4.3 (4.1–4.5)	4.6 (4.4–4.8)	4.5 (4.2–4.7)	<0.001	<0.001
Albumin < 3.5 g/dL, n (%)	2 (3.8)	0 (0)	0 (0)	0.157	-
INR	1.1 (1.0–1.2)	1.0 (1.0–1.1)	1.0 (1.0–1.1)	<0.001	0.101
INR > 1, n (%)	46 (76.8)	43 (82.7)	32 (62.8)	0.564	0.018
Platelets (10^9^/L)	141 (103–180)	173 (128–206)	173 (137–225)	<0.001	0.008
Platelets < 150 (10^9^/L)	28 (52.8)	19 (36.5)	21 (38.9)	0.011	0.480
Splenomegaly (>12 cm), n (%)	25 (47.2)	14 (31.1)	8 (14.8)	0.034	0.020
Oesophageal varices, n (%) ^1^	15 (27.8)	7 (13.0)	9 (16.7)	0.083	0.317
VCTE-LSM (kPa)	16.2 (12.0–21.8)	9.9 (7.8–13.4)	9.7 (7.5–13.6)	<0.001	0.386
Group C, n (%)	20 (37.0)	76(12.2)	8 (14.8)	0.001	0.527
Group B, n (%)	10 (18.5)	9 (18.4)	9 (16.7)		
Group A n (%)	24 (44.4)	34 (69.4)	37 (68.5)		
<10 kPa, (n%)	-	25 (51.0)	31 (57.4)	-	0.285
<8 kPa, n (%)	-	13 (26.5)	17 (31.5)	-	0.257
<6 kPa	-	2 (4.1)	5 (9.3)	-	0.180
Baveno VI/VII criteria, n (%)				0.011	0.854
Group C	32 (59.3)	21 (41.2)	23 (42.6)		
Group B	4 (7.4)	5 (9.8)	3 (5.6)		
Group A	18 (33.3)	25 (49.0)	28 (51.9)		

BMI: body mass index. T2DM: type 2 diabetes mellitus. ALT: alanine-aminotransferase. INR: international normalized ratio. VCTE-LSM: liver stiffness measurement by vibration-controlled transient elastography. Statistical comparisons between before EOT and 3 y after EOT (*p*^1^), and between 3 y post-EOT and 6 y post-EOT (*p*^2^). ^1^ Upper gastrointestinal endoscopy was available in 34 patients before EOT, in 14 at 3 years after EOT, and in 16 at 6 years after EOT. In the remaining patients, the absence of varices was assumed based on non-invasive testing.

**Table 2:** Histological characteristics of liver biopsies at 3 and 6 years post-EOT.

	All Patients		Paired Biopsies		
	LB3 (n = 34)	LB6 (n = 54)	LB3 (n = 34)	LB6 (n = 34)	*p* Paired
Time from EOT (months)	38.0 (26.6–40.9)	81.4 (72.5–87.3)	38.0 (26.6–40.9)	84.2 (79.5–87.8)	-
Length (mm)	31 (21–34)	32 (26–35)	31 (21–34)	32 (26–35)	0.289
Portal spaces, n (%)	11 (7–24)	18 (9–23)	11 (7–24)	18 (9–21)	0.480
METAVIR hepatitis activity, n (%)					1.000
A0–1	34 (100)	52 (96.3)	34 (100)	34 (100)	
A2	0 (0)	2 (3.7)	0 (0)	0 (0)	
NASH CRN steatosis, n (%)					0.052
<5%	25 (73.5)	28 (51.9)	25 (73.5)	20 (58.8)	
5–30%	8 (23.5)	23 (42.6)	8 (23.5)	11 (32.4)	
30–60%	1 (2.9)	3 (5.6)	1 (2.9)	3 (8.8)	
METAVIR fibrosis stage, n (%)					0.029
F1–F2	8 (23.5)	12 (22.2)	8 (23.5)	8 (23.5)	
F3	7 (20.6)	26 (48.2)	7 (20.6)	16 (47.1)	
F4	19 (55.9)	16 (29.6)	19 (55.9)	10 (29.4)	
Laennec fibrosis stage, n (%)					0.679
F4A	13 (38.2)	7 (13.0)	13 (38.2)	5 (14.7)	
F4B	4 (11.8)	4 (7.4)	4 (11.8)	2 (5.9)	
F4C	2 (5.9)	5 (9.3)	2 (5.9)	3 (8.8)	

LB3: liver biopsy at 3 years post-EOT. LB6: liver biopsy at 6 years post-EOT. EOT: end of treatment.

**Table 3:** Differences between patients according to liver fibrosis 6 years after EOT.

	Liver Fibrosis 6 Years After EOT			
	METAVIR F1–F2	METAVIR F3	METAVIR F4	*p*
Before DAA (n = 54)	(n = 12)	(n = 26)	(n = 16)	F1–F2 vs. F3–F4
Age (years)	56.0 (53.3–60.3)	51.3 (47.3–58.3)	53.1 (49.4–58.3)	0.075
Male, n (%)	10 (83.3)	16 (61.5)	12 (75.0)	0.265
BMI (kg/m^2^)	26.4 (23.7–27.1)	27.4 (24.0–30.5)	26.9 (24.8–33.5)	0.359
T2DM, n (%)	1 (8.3)	4 (15.4)	5 (31.3)	0.303
Arterial hypertension, n (%)	2 (16.7)	8 (30.8)	2 (12.5)	0.600
Alcohol abuse, n (%)	2 (16.7)	3 (11.5)	1 (6.3)	0.487
HIV coinfection, n (%)	1 8.3)	6 (23.1)	6 (37.5)	0.148
HCV genotype 3, n (%)	1 (8.3)	9 (34.6)	4 (25.0)	0.115
ALT (U/L)	55 (42–67)	62 (46–114)	60 (45–99)	0.167
Bilirubin (mg/dL)	0.7 (0.5–0.9)	0.8 (0.6–1.0)	1.0 (0.7–1.6)	0.076
INR	1.0 (1.0–1.1)	1.1 (1.0–1.1)	1.2 (1.1–1.3)	0.012
Albumin (g/dL)	4.5 (4.4–4.6)	4.3 (4.1–4.5)	4.2 (4.0–4.5)	0.031
Platelets (10^9^/L)	186 (162–214)	141 (91–174)	108 (90–140)	0.002
Platelets < 150 (10^9^/L)	2 (16.7)	13 (52.0)	13 (81.3)	0.004
Splenomegaly (>12 cm), n (%)	2 (16.7)	14 (56.0)	9 (56.3)	0.016
Oesophageal varices, n (%)	0 (0)	9 (34.6)	6 (37.5)	0.015
VCTE-LSM (kPa)	10.5 (10.1–11.9)	15.4 (14.0–20.9)	23.2 (18.6–31.0)	<0.001
>20 kPa, n (%)	0 (0)	9 (34.6)	11 (68.8)	0.001
15–20 kPa, n (%)	1 (8.3)	6 (23.1)	3 (18.8)	
<15 kPa, n (%)	11 (91.7)	11 (42.3)	2 (12.5)	
Baveno VI criteria, n (%)				<0.001
LSM > 20 kPa and/or PLT < 150·(10^9^/L)	2 (16.7)	15 (57.7)	15 (93.8)	
LSM 15–20 kPa and PLT > 150·(10^9^/L)	0 (0)	3 (11.5)	1 (6.2)	
LSM < 15 kPa and PLT > 150·(10^9^/L)	10 (83.3)	8 (30.8)	0 (0)	
1 year after EOT (n = 51)	(n = 10)	(n = 25)	(n = 16)	
Platelets (10^9^/L) (n = 45)	206 (180–240)	179 (132–205)	130 (114–170)	0.016
Platelets < 150 (10^9^/L)	1 (10.0)	7 (31.8)	7 (53.9)	0.016
VCTE-LSM (kPa)	7.5 (6.3–8.6)	11.9 (10.5–14.0)	20.1 (12.1–24.8)	<0.001
>20 kPa, n (%)	0 (0)	4 (16.0)	8 (50.0)	0.006
12–20 kPa, n (%)	0 (0)	7 (28.0)	4 (25.0)	
<12 kPa, n (%)	10 (100)	14 (56.0)	4 (25.0)	
<10 kPa, n (%)	9 (90.0)	6 (24.0)	1 (6.3)	<0.001
<8 kPa, n (%)	7 (70.0)	1 (4.0)	0 (0)	<0.001
Baveno VII criteria, n (%) (n = 48)				
LSM > 20 kPa and/or PLT < 150·(10^9^/L)	1 (11.1)	10 (43.5)	12 (75.0)	0.001
LSM 12–20 kPa and PLT > 150·(10^9^/L)	0 (0)	6 (26.1)	2 (12.5)	
LSM < 12 kPa and PLT > 150·(10^9^/L)	8 (88.9)	7 (30.4)	2 12.5)	
3 years after EOT (n = 34)	(n = 8)	(n = 16)	(n = 10)	
METAVIR hepatitis activity, n (%)				0.722
A0/A1	3 (37.5)/5 (62.5)	4 (25.0)/12 (75.0)	4 (40.0)/6 (60.0)	
NASH CRN steatosis, n (%)				0.914
<5%/>5%	6 (75.0)/2 (25.0)	11 (68.8)/5 (31.2)	8 (80.0)/2 (20.0)	
METAVIR fibrosis stage, n (%)				0.006
F1–F2	5 (62.5)	3 (18.8)	0 (0)	
F3	2 (25.0)	5 (31.3)	0 (0)	
F4	1 (12.5)	8 (50.0)	10 (100)	
Laennec fibrosis stage, n (%)				0.784
F4A	1 (12.5)	7 (43.8)	5 (50.0)	
F4B	0 (0)	1 (6.3)	3 (30.0)	
F4C	0 (0)	0 (0)	2 (20.0)	

F1–F2: non-advanced fibrosis. F3: bridging fibrosis. F4: cirrhosis (F4). BMI: body mass index. T2DM: type 2 diabetes mellitus. ALT: alanine-aminotransferase. INR: international normalized ratio. VCTE-LSM: liver stiffness measurement by vibration-controlled transient elastography.

## Data Availability

The original contributions presented in this study are included in the article. Further inquiries can be directed to the corresponding author.

## Abbreviations

The following abbreviations are used in this manuscript:

ALT	Alanine-aminotransferase
AUROC	Area under the receiver operating characteristics
BMI	Body mass index
cACLD	Compensated advanced chronic liver disease
CSPH	Clinically significant portal hypertension
DAA	Direct-acting antivirals
EOT	End of treatment
HCC	Hepatocellular carcinoma
HCV	Hepatitis C virus
HIV	Human immunodeficiency virus
INR	International normalized ratio
IQR	Interquartile range
LB3	Liver biopsy 3 years after EOT
LB6	Liver biopsy 6 years after EOT
LREs	Liver-related events
NITs	Non-invasive tests
PLT	Platelet count
T2DM	Type 2 diabetes mellitus
SVR	Sustained virological response
VCTE-LSM	Liver stiffness measurement by vibration-controlled transient elastography
